# The diversification of species in crop rotation increases the profitability of grain production systems

**DOI:** 10.1038/s41598-022-23718-4

**Published:** 2022-11-18

**Authors:** Bruno Volsi, Gabriel Eiji Higashi, Ivan Bordin, Tiago Santos Telles

**Affiliations:** 1 Department of Agronomy, Universidade Estadual de Londrina, Rodovia Celso Garcia Cid, Km 380, Londrina, Paraná 86057-970 Brazil; 2 Instituto de Desenvolvimento Rural do Paraná – IAPAR-EMATER, Rodovia Celso Garcia Cid, Km 375, Londrina, Paraná 86047-902 Brazil

**Keywords:** Environmental economics, Environmental economics, Sustainability, Ecosystem services, Agroecology

## Abstract

Crop rotation with species diversification contributes to the control of pests, diseases and weeds and improves soil fertility and conservation, which can lead to increased profitability in grain production systems. The objectives of this study were to determine whether grain production systems that employ crop rotation with species diversification are more productive and profitable than double-cropping rotations without diversification and to analyze the revenues and production costs of these cropping systems. An experiment was conducted in a region with subtropical climate between the crop years of 2014–2015 and 2019–2020. The experiment consisted of a randomized block design with six treatments and four replicates. The treatments consisted of six grain production systems, including five rotations with varied levels of species diversification and a corn–soybean rotation without species diversification, all under no-tillage. Productivity, revenue, production cost and profit indicators were analyzed. Productivity was compared by Duncan’s test (*p* ≤ 0.05). The grain production systems with species diversification showed better productivity and profitability than the corn–soybean system. The profit of the systems with species diversification was on average 37% higher than that of the system with corn–soybean rotation. In summary, grain production systems that employ crop rotation with species diversification showed higher productivity and profitability than the corn–soybean rotation without species diversification.

## Introduction

In the tropical and subtropical regions of Brazil, double cropping or planting two crops in one crop year is a common system under no-tillage. Double cropping became predominant in grain production in the country, especially with corn as the second crop in the autumn/winter followed by soybean in the summer. This soybean–corn rotation has been a relatively successful system but has caused concern because the principles of neither no-tillage systems nor conservation agriculture are followed. These principles, established in the 1970s and 1980s^1,2^, call for crop rotation with species diversification to maintain ground cover and enhance soil health.

The main problems resulting from double cropping include increased incidence and resistance of pests and diseases^3^, weed infestation^4^, reduced soil fertility, especially of organic matter^5,6^, soil compaction^7^ and soil loss due to water erosion^8^. In addition, climate change, together with droughts and abrupt temperature changes, has increased the production risks in double-crop systems^9,10^, as they become less resistant to environmental shocks and more susceptible to production losses. Thus, maintaining or achieving higher productivity and profitability levels becomes a challenge for farmers who adopt double cropping^11,12^. To avoid the problems encountered in double cropping, which may compromise the sustainability of agricultural production systems, species diversification through crop rotation is an efficient solution and is considered sustainable^13^ for intensifying grain production in Brazil.

Crop rotation, when performed continuously, can improve physical, chemical and biological soil attributes, especially by increasing the content of organic carbon^14^, nitrogen^15^ and nutrients readily available to plants^16^. These benefits depend on the crop species and cropping sequences adopted by the producer, and commercial plants combined, whenever possible, with regionally adapted, fast-growing cover crops able to produce large amounts of dry matter are recommended^17,18^.

Compared with the double-crop system, crop rotation increases resistance and resilience to abiotic stresses and improves crop productivity throughout the rotation cycles^19^. Thus, diversification of crop rotations can ensure the conservation of natural resources and reduce production risks. Although crop rotations have numerous benefits, some farmers in Brazil still opt for the double-cropping system for grain production due to the lack of short-term economic benefits.

Although the agronomic benefits of crop rotation practices are well described in the literature, studies reporting their economic advantages are still scarce^20–24^. Research on the economic results of grain production systems in Brazil is of great relevance, as it helps and facilitates the decision-making of farmers. Therefore, the hypothesis of this study is that grain production from rotations with species diversification will result in better productivity and profitability than that from double-crop rotations without species diversification. In this context, the objectives of this study were to determine whether grain production systems that employ crop rotation with species diversification are more productive and profitable than double-cropping rotations without diversification and to analyze the revenues and production costs of these systems.

## Materials and methods

### Experimental site

The experiment was conducted at the Institute of Rural Development of Paraná-IAPAR-EMATER (IDR-Paraná, its acronym in Portuguese), located in the municipality of Londrina, Paraná State, southern Brazil, at geographic coordinates 23° 22’S and 51° 10’W, at 585 m altitude, in Typic Eutroferric Red Latosol with clayey texture, and on smooth undulating terrain^25^.

The study comprises six crop years, from 2014–2015 to 2019–2020, subdivided into two three-year cycles. The first cycle encompassed the 2014–2015 to 2016–2017 crop years, and the second the 2017–2018 to 2019–2020 crop years. Two annual harvests were performed, i.e., winter and summer. The winter harvest corresponds to crops whose growing cycle occurred in fall-winter, while the summer harvest corresponds to crops whose growing cycle occurred in spring–summer.

**Figure 1:**
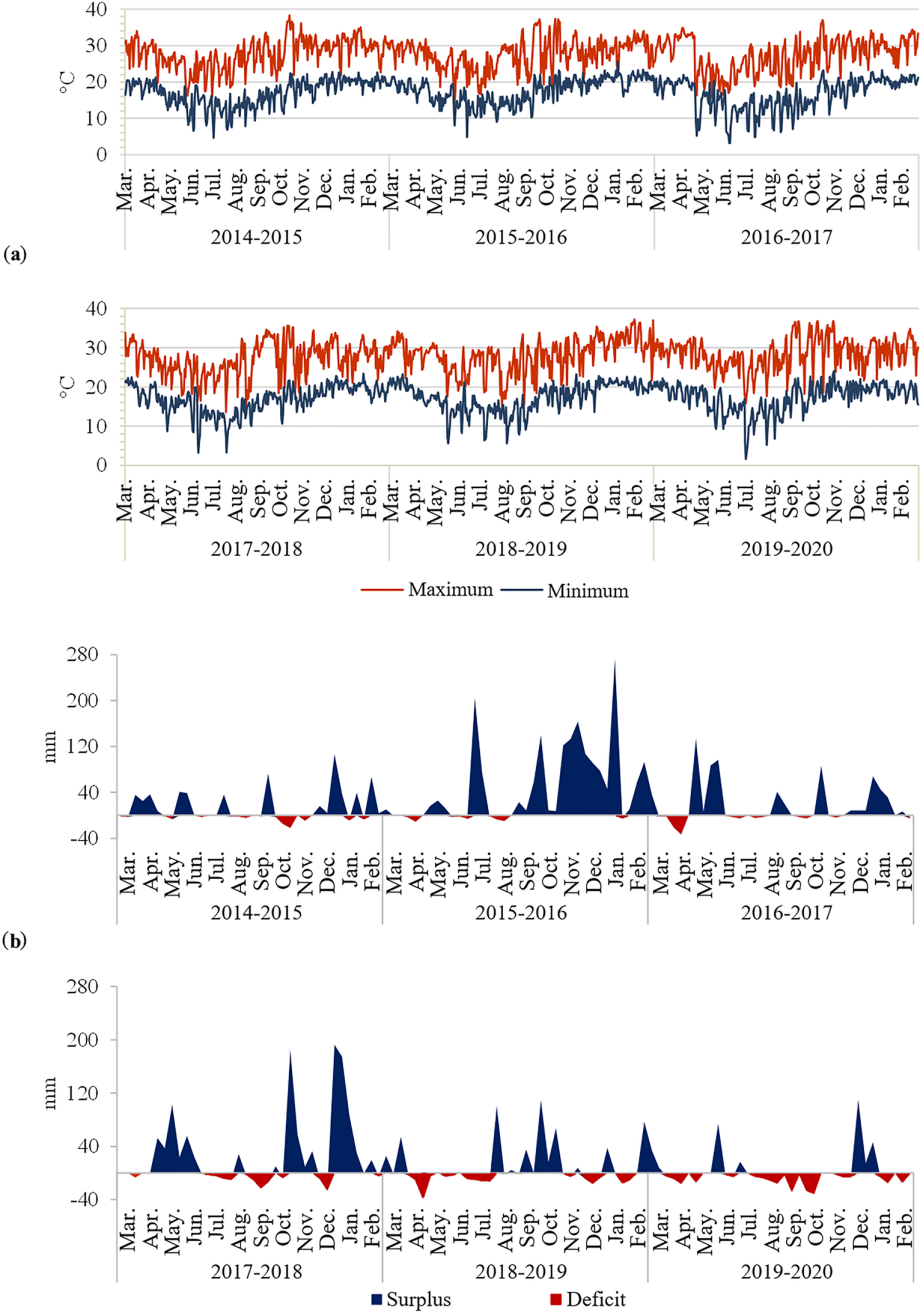
(**a**) Maximum and minimum daily temperatures and (**b**) ten-year water balance (AWC = 80 mm), Londrina, Paraná State, Brazil, between crop years 2014–2015 and 2019–2020. Prepared based on data from the Paraná Environmental Technology and Monitoring System (Sistema de Tecnologia e Monitoramento do Paraná—SIMEPAR).

According to Köppen^26^, the climate of the region is subtropical (Cfa), characterized by hot and humid summers, with a mean annual temperature of 21.2 °C and mean annual rainfall of 1632 mm. To characterize the climatic conditions of the site and years in which the study occurred, data on maximum and minimum daily temperatures, rainfall, and available water capacity were collected to calculate the ten-year water balance (Fig. 1).

### Experimental design

The experimental design used was randomized blocks consisting of six treatments and four replicates. The treatments consisted of one double-crop system (corn–soybean) and five crop rotation systems with different levels of species diversification (Table 1). Agricultural system I (AS-I), considered nondiversified, consisted of double cropping of corn (*Zea mays* L.) in the winter, followed by soybean (*Glycine max* (L.) Merr.) in the summer and is adopted by most grain producers. Agricultural system II (AS-II) included a larger number of winter cereal species, and in this system, the corn produced in winter was replaced with white oat (*Avena sativa* L.), rye (*Secale cereale* L.), triticale (× *Triticosecale* Wittm. ex A. Camus) and, in the last harvest, wheat (*Triticum aestivum* L.). Regarding agricultural system III (AS-III), the winter crops were cover crops, including black oat (*Avena strigosa* Schreb.), brachiaria (*Brachiaria ruziziensis Germ. & Evrard*) and rye, in addition to forage radish (*Raphanus sativus* L.). Agricultural system IV (AS-IV) focused on agroenergy and produced corn and soybean in the summer and canola, crambe (*Crambe abyssinica* Hochst. ex R.E. Fries) and safflower (*Carthamus tinctorius* L.) in the winter (oilseed crops) and was one of the more diversified systems. In agricultural system V (AS-V), commercial crops such as bean (*Phaseolus vulgaris* L.) and crops with commercial potential, such as buckwheat (*Fagopyrum esculentum* Moench), were grown in the winter. Agricultural system VI (AS-VI) was one of the most diversified crop rotation systems, with the cultivation of six species: wheat, corn, brachiaria, canola, beans and soybean.

**Table 1:** Grain production systems and sowing dates in winter and summer in two agricultural cycles, between the crop years 2014–2015 and 2019–2020, in Londrina, state of Paraná, Brazil.

1st cycle						2nd cycle					
2014–2015		2015–2016		2016–2017		2017–2018		2018–2019		2019–2020	
Winter	Summer	Winter	Summer	Winter	Summer	Winter	Summer	Winter	Summer	Winter	Summer
**System I (AS-I)**											
C	S	C	S	C	S	C	S	C	S	C	S
03/2014^a^	10/2014	03/2015	10/2015	03/2016	10/2016	03/2017	10/2017	03/2018	10/2018	03/2019	10/2019
**System II (AS-II)**											
WO	S	RY	C	W	S	WO	S	TR	C	W	S
05/2014	10/2014	04/2015	10/2015	05/2016	10/2016	04/2017	10/2017	05/2018	10/2018	04/2019	10/2019
**System III (AS-III)**											
BO + RY	S	BO + R	C	BR	S	BO + RY	S	BO + R	C	BR	S
05/2014	10/2014	04/2015	10/2015	03/2016	10/2016	04/2017	10/2017	05/2018	10/2018	03/2019	10/2019
**System IV (AS-IV)**											
CL	C	CM	C	SF	S	CL	C	CM	C	CL	S
05/2014	10/2014	04/2015	10/2015	03/2016	10/2016	05/2017	10/2017	04/2018	10/2018	04/2019	10/2019
**System V (AS-V)**											
BW/R	C	B	S	BW/WO	S	BW/R	C	B	S	BW/WO	S
03/2014	10/2014	04/2015	10/2015	03/2016	10/2016	03/2017	10/2017	04/2018	10/2018	03/2019	10/2019
**System VI (AS-VI)**											
W	C + BR	CL	C	B	S	W	C	CL	C + BR	B	S
06/2014	10/2014	05/2015	10/2015	03/2016	10/2016	04/2017	10/2017	05/2018	10/2018	03/2019	10/2019

*WO* white oat, *BO* black oat, *BR* brachiaria, *CL* canola, *CM* crambe, *RY* rye, *SF* safflower, *B* bean, *C* corn, *R* forage radish, *S* soybean, *W* wheat, *BW* buckwheat, *TR* triticale.

^a^The information refers to the planting dates of each crop.

In the second cycle of AS-II, in the winter of the 2018–2019 crop year, rye was replaced by triticale (Poaceae) with the objective of producing grains. In AS-IV, safflower was replaced by canola in the winter of 2019–2020. Although the species are from different families, both crops are oilseeds that serve the same purpose. In AS-VI, unlike the first cycle, the corn-brachiaria intercrop was grown in the summer of the second rotation year, in the 2018–2019 crop year. This was due to the high production of straw in the consortium, making the canola sowing operation challenging in the following harvest. Thus, its cultivation was carried out before bean cultivation, and the dynamics of the system were not altered.

The area of each experimental plot measured 15 × 20 m (300  m^2^), with a spacing of 10 m between the plots for the maneuvering of agricultural machinery and implements. Prior to the start of the experiment, the area was under no-tillage for 12 years, with alternating cultivation of black oat in the winter and corn and soybean in the summer.

### Economic analysis

For the economic evaluation, the revenue-to-cost ratio of each production system was used, with the profit as the result. The revenues were calculated based on the mean production obtained in each treatment multiplied by the sale price on the harvest date. Productivity, in turn, was obtained by weighing the harvested grains from the useful area of the plots, corrected for 13% moisture (wet basis), and the values were expressed in kg ha^−1^. To calculate the cost, all the services and inputs used to finance the production systems were considered. The price of the agricultural operations (manual and mechanical) and the inputs used are presented in dollars per hectare (US$ ha^−1^). To calculate the costs, other expenses inherent to production were also considered, such as external transportation, labor, land tax and technical assistance. Profit was obtained by the difference between revenue and production cost.

To obtain the costs of both agricultural operations and inputs, a survey was conducted on the mean prices paid and received by producers in July of each crop year, i.e., from 2014–2015 to 2019–2020. Supplementary Table S1 shows the grain prices paid each year of the study.

To convert nominal values into real values, all economic indicators were corrected to December 2021 values using the Extended National Consumer Price Index (IPCA, acronym in Portuguese), which is the official inflation index in Brazil. The real values were converted from Brazilian Real (BRL) to US dollars (USD) using the conversion rate for December 31, 2021 (1 USD = BRL 5.58).

### Sensitivity analysis

To verify the effect of changes in the sale prices of the main crops sold (soybean, corn, wheat and beans) on the profit of production systems, a sensitivity analysis was performed on the fluctuations in retained earnings due to percentage changes from the actual sales prices (every 1%, up to 50% higher and 50% lower). With this method, we measure the impact of the change in the real selling price^27^ on the accumulated profit by every 1% in each year. This procedure was repeated for the main crops sold in the systems.

### Statistical analysis

To verify the effect of the agricultural production systems, statistical analysis was performed using SAS On Demand for Academics (SAS Institute, Inc., Cary, NC) at the end of the first cycle (third crop year) and at the end of the second cycle (sixth crop year), where all the systems had the same crop. The data for the 2016–2017 and 2019–2020 crop years were subjected to analysis of variance, and the means were compared by Duncan’s test (*p* ≤ 0.05).

To determine the aboveground dry matter of all the crops at physiological maturity, four samples 0.25 m^2^ were collected per plot and dried in a forced ventilation oven at 60 °C for 72 h. The data were subjected to analysis of variance, and means were compared by Tukey's test at 5% significance.

## Results and discussion

### Productivity

With regard to productivity, in the summer harvest of the 2016–2017 crop year, in which all grain production systems had soybean in common, there were significant differences among crop rotations with species diversification and the double-cropped corn–soybean rotation; performance was better in AS-II, AS-III, AS-IV, AS-V and AS-VI and worst in AS-I. There was no significant difference in productivity among the crop rotations with species diversification (Table 2).

**Table 2:** Productivity (kg ha^−1^) of the crop rotation systems for the 2014–2015 to 2019–2020 crop years in Londrina, state of Paraná, Brazil.

1st cycle						2nd cycle					
2014–2015		2015–2016		2016–2017		2017–2018		2018–2019		2019–2020	
Winter	Summer	Winter	Summer	Winter	Summer	Winter	Summer	Winter	Summer	Winter	Summer
**System I (AS-I)**											
C	S	C	S	C	S	C	S	C	S	C	S
5895	2965	3536	3241	2737	4030 b	8040	4139	5775	3285	5340	3625 b
**System II (AS-II)**											
WO	S	RY	C	W	S	WO	S	TR	C	W	S
3247	3518	–	9867	3762	4488 a	1516	4790	3401	8802	3842	3864 ab
**System III (AS-III)**											
BO + RY	S	BO + R	C	BR	S	BO + RY	S	BO + R	C	BR	S
–	3431	–	10,300	–	4591 a	–	4231	–	8761	–	3848 ab
**System IV (AS-IV)**											
CL	C	CM	C	SF	S	CL	C	CM	C	CL	S
1756	11,202	1475	9252	1006	4397 a	367	10,650	–	8630	1084	4027 a
**System V (AS-V)**											
BW/R	C	B	S	BW/WO	S	BW/R	C	B	S	BW/WO	S
1161	10,630	1984	3006	785	4369 a	1654	11,502	1851	3448	1688	3650 b
**System VI (AS-VI)**											
W	C + BR	CL	C	B	S	W	C	CL	C + BR	B	S
1833	10,532	1142	9591	776	4617 a	4046	10,340	1282	7915	2337	4068 a

*WO* white oat, *BO* black oat, *BR* brachiaria, *CL* canola, *CM* crambe, *RY* rye, *SF* safflower, *B* bean, *C* corn, *R* forage radish, *S* soybean, *W* wheat, *BW* buckwheat, *TR* triticale.

“– “: no grain production took place. The data for the summer of the 2016–2017 and 2019–2020 crop years were subjected to analysis of variance according to Duncan’s test (*p* ≤ 0.05).

Means followed by the same letter in a column do not differ statistically according to Duncan's test at 5% probability.

For the summer harvest of the 2019–2020 crop year, in which all the grain production systems again had soybean in common, significant differences were also observed among the production systems. AS-I and AS-V had the lowest productivities, differing from AS-IV and AS-VI, which had the highest productivities. Conversely, the productivities of AS-II and AS-III did not differ significantly from those of the other evaluated systems (Table 2).

In the cycle that ended in crop year 2019–2020, compared to the cycle that ended in crop year 2016–2017, there was a reduction in soybean productivity in all the analyzed grain production systems (Table 2). There was also a decrease in the productivity of corn grown in the summer in the 2015–2016 and 2018–2020 crop years. This decrease in productivity observed between the production cycles may be associated with climatic conditions because from 2014–2015 to 2016–2017, there was a good rainfall distribution and few water deficit peaks, while from 2017–2018 to 2019–2020, the water deficit peaks were more constant, especially in 2018–2019 and 2019–2020 (Fig. 1). Notably, there was a greater influence of the El Niño phenomenon on the first production cycle (2014–2017) and of the La Niña phenomenon on the second (2017–2020)^28^. In southern Brazil, these phenomena correspond to periods of weaker droughts under El Niño conditions and a higher frequency of severe and moderate droughts under La Niña conditions^29^. The occurrence of a water deficit may limit plant growth and development, particularly during the flowering and grain filling stages. Systems that employ crop rotation with species diversification are less susceptible to production losses due to water deficits^30^. The results of this study show that crop rotation systems with species diversification, by providing a longer soil cover time for soil protection, either with live plants or from the input of surface straw, together with the respective increase in the soil water storage capacity, can mitigate productivity losses resulting from periods of drought (Fig. 1, Table 2).

Another finding is that soybean has higher productivity when grown in systems with greater species diversification, as was the case for AS-IV and AS-VI (Table 2). In general, grain production systems that employ crop rotation with species diversification produce more than those that are not diversified^31,32^, especially in atypical growing seasons affected by climatic factors limiting crop development^33^.

AS-I and AS-V showed the lowest soybean productivity at the end of the second crop rotation cycle, in the 2019–2020 crop year (Table 2). AS-I had the lowest soybean productivity at the end of the two crop cycles, i.e., in 2016–2017 and 2019–2020, a result that is directly related to corn–soybean double cropping. In the southern region of Brazil, for example, soybean productivity in crop rotation systems with species diversification is 6.2% higher than that in double-crop systems^22^. In this sense, the results of this study indicate that production systems with little species diversification have lower soybean productivity than those that employ crop rotation with species diversification.

At the end of the second crop rotation cycle, in 2019–2020, AS-II and AS-III also showed good soybean productivity, i.e., 3864 kg ha^−1^ and 3848 kg ha^−1^, respectively. AS-III had one of the highest grain yields in the summer crops, which may be associated with the use of cover crops in the previous winter. The use of cover crops in the winter growing seasons results in a number of benefits from permanent soil cover because cover crops can improve chemical, physical and biological soil attributes, favoring the accumulation of biomass and organic carbon in the soil^34^ and prevent soil erosion^35^. In addition, cover crops control pests, diseases and weeds^36^ and contribute to weed^37^ and nematode^38^ control.

Regarding crop dry matter, AS-III, AS-IV, AS-V and AS-VI (Table 3) deposited the most dry matter in the system; the crop dry matter in these systems was greater than that in AS-I and showed no significant difference in relation to that in AS-II. The lower production of dry matter in AS-I is explained by the lack of corn cultivation in the summer. Corn grown in the summer was the crop that most contributed to the accumulation of dry matter in AS-III, AS-IV and AS-VI, compensating for the low averages obtained with beans in AS-V and AS-VI and with safflower in AS-IV. The higher dry matter inputs in AS-IV and AS-VI are because these are the only systems in which corn was grown in the summer for two consecutive years. The average dry matter contributed by corn grown in the summer is 9.9 Mg ha^−1^, while that from off-season corn and soybeans is 6.5 Mg ha^−1^ and 4.35 Mg ha^−1^, respectively.

**Table 3:** Dry matter (Mg ha^−1^) of the grain production systems for the 2014–2015 to 2019–2020 crop years in Londrina, state of Paraná, Brazil.

1st cycle						2nd cycle						Annual mean
2014–2015		2015–2016		2016–2017		2017–2018		2018–2019		2019–2020		
W	S	W	S	W	S	W	S	W	S	W	S	
**System I (AS-I)**												
C	S	C	S	C	S	C	S	C	S	C	S	
7.6	5.10	6.9	6.0	5.3	4.8	8.1	3.5	5.7	3.30	5.6	3.9	10.98 b
**System II (AS-II)**												
WO	S	RY	C	W	S	WO	S	TR	C	W	S	
5.6	4.46	7.2	9.1	5.2	4.7	6.9	3.0	4.9	8.10	6.1	4.1	11.56 ab
**System III (AS-III)**												
BO + RY	S	BO + R	C	BR	S	BO + RY	S	BO + R	C	BR	S	
8.2	4.72	6.1	10.6	7.8	4.0	5.7	4.2	4.2	7.30	6.6	3.8	12.11 a
**System IV (AS-IV)**												
CL	C	CM	C	SF	S	CL	C	CM	C	CL	S	
7.7	12.0	4.1	11.5	4.0	4.6	3.3	11.0	–	7.20	5.0	4.0	12.40 a
**System V (AS-V)**												
BW/R	C	B	S	BW/WO	S	BW + R	C	B	S	BW/WO	S	
12.7	10.9	2.3	5.6	5.7	5.3	7.3	11.9	1.7	3.80	2.3	3.9	12.24 a
**System VI (AS-VI)**												
W	C + BR	CL	C	B	S	W	C	CL	C + BR	B	S	
3.8	12.5	4.8	9.2	1.7	4.5	5.9	10.6	4.3	11.30	2.2	4.1	12.50 a

*W* winter, *S* summer, *WO* white oat, *BO* black oat, *BR* brachiaria, *CL* canola, *CM* crambe, *RY* rye, *SF* safflower, *B* bean, *C* corn, *R* forage radish, *S* soybean, *W* wheat, *BW* buckwheat, *TR* triticale

Means followed by the same letter in a column do not differ statistically according to Duncan's test at 5% probability.

Studies carried out in the Cerrado, Mato Grosso, showed that the minimum amount of plant dry matter deposited by crop rotation systems needed to obtain a balance of C in the soil in the region is between 11.7 and 13.3 Mg ha^−1^^39^. Therefore, we can deduce that AS-III, AS-IV, AS-V and AS-VI would enter equilibrium; that is, over time, there will be neither accumulation of nor loss of C from the soil. For AS-I and AS-II, we can conclude that over time, C stocks in the soil will be reduced, causing a loss of soil fertility and, consequently, productivity, as shown in Table 2, where the yield of AS-I was lower than that of the most diversified treatments.

The results show that crop diversification in grain production systems with the cultivation of commercial or cover crops in the winter benefited soybean and corn production in the summer. In similar studies, species diversification is reported to have increased summer crop productivity over time; specifically, in the U.S. and Canada, corn productivity increased by an average of 28.1%^40^, and in Canada, corn yield increased by 9.9% and soybean productivity increased by 11.8%^41^.

### Economic analysis

**Figure 2:**
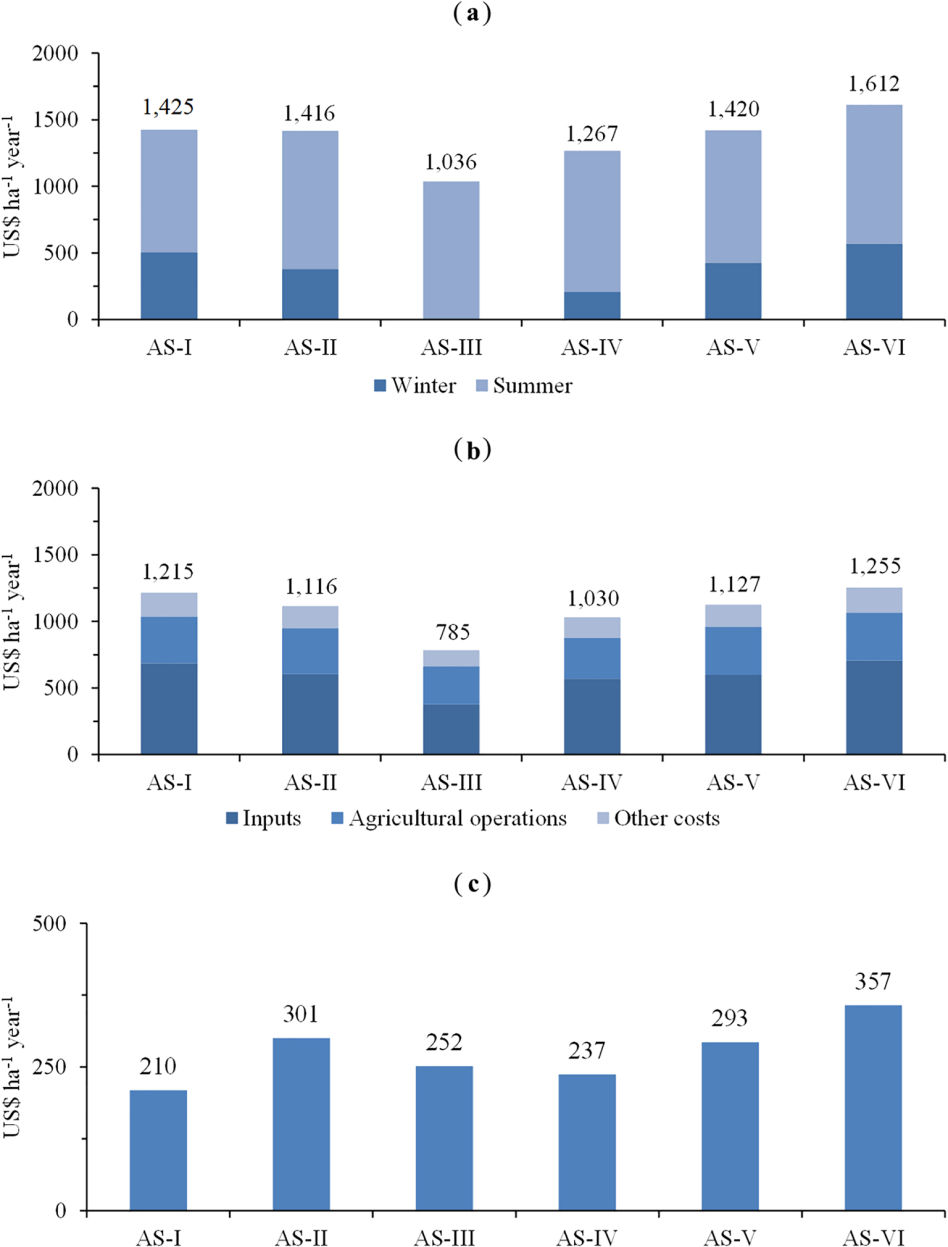
(**a**) Mean annual revenue, (**b**) mean annual cost and (**c**) mean annual profit of grain production systems with varied levels of species diversity in Londrina, state of Paraná, Brazil.

The highest mean annual revenue was found for AS-VI, while the lowest was found for AS-III. Regarding the mean annual cost, AS-VI demanded the greatest investment, while AS-III showed the lowest production cost. The highest mean annual profit was also observed for AS-VI, highlighting that the revenue more than offset the costs. As expected, the lowest mean annual profit was found for AS-I, that is, the corn–soybean double-crop system (Fig. 2).

The higher profitability observed for AS-VI indicates that the practice of crop rotation with species diversification in grain production systems increased the grain productivity and economic gains. In this system, the productivity of the commercial crops was positively impacted, and the crops showed excellent yields compared to those in the production systems with lower species diversification. In addition, the winter crops played a key role in the composition of the revenues, especially wheat and bean. As previously noted, the highest mean annual costs of inputs (US$ 685), agricultural operations (US$ 353) and other costs (US$ 177) were found for this system. Within the inputs, the highest cost was for fertilizers (K_2_O, P_2_O_5_, and N), accounting for approximately 22% of the total cost (US$ 280). The higher cost may be related to higher energy demands because in a grain production system, a greater energy volume represents a greater use of inputs^42^. However, although the cost was the highest, the system was found to be more capable of converting investments into higher productivity and, consequently, into higher revenue and profit. Other studies conducted in Brazil also found economic benefits in crop rotation systems with species diversification, for example, in areas with a predominance of Caiuá sandstone, a region with low-fertility soils, in which the highest profitability was obtained in diversified systems that adopted the highest number of commercial crops, both in the winter and summer growing seasons^21^. Similarly, in another study in southern Brazil, higher productivities were obtained for more diversified crop rotation systems^23^. In a long-term study involving soybean, corn, wheat and tropical forage grasses in southern Brazil, higher profits were also found for more diversified production systems^22^.

AS-II had the second highest mean annual profit; this system is characterized by the cultivation of cereals in the winter. The results show that this grain production system is promising, as the use of winter cereal crops had a positive effect on the productivity of the summer crops, leading to increased revenue and profit from the sale of soybean and corn (Supplementary Table S2). With regard to costs, the items that generated the highest expenses in AS-II were inputs, accounting for an average of 54% of the total cost, followed by agricultural operations, which represented an average of 31% of the total, and other costs, accounting for an average of 15% of the total cost (Supplementary Table S2). Studies conducted in other locations also recommend crop rotation systems with the use of cereals, as in the semiarid Northern Great Plains, Canada, where higher productivity and greater profit were found with these cultivation systems compared to a system without species diversification^43^.

AS-V had the third highest mean annual profit. This system is composed of six different crops, and its profitability results were also relevant. Regarding the revenues obtained in the winter growing seasons, beans stood out, accounting for 21% of the total (Supplementary Table S2). One of the problems with AS-V was the cultivation of buckwheat, which, in addition to having a low market price and generating little revenue, also had a high production cost, negatively impacting the entire production system. Thus, if buckwheat had not been cultivated, AS-V could have achieved higher profitability than that observed. With regard to the costs for AS-V, the cost of inputs represented an average of 53% of the total cost, followed by agricultural operations (on average, 31% of the total cost) and other costs (on average, 15% of the total). The cultivation of legumes such as beans in the winter is beneficial for grain production systems because it can favor increased production and, consequently, the profit obtained with subsequent crops^44^.

AS-III had the fourth highest mean annual profit. Although this system did not have the best profitability, it should not be disregarded. This system is focused on the production of straw in the winter and on the revenue generated by the summer crops. However, although cover crops do not generate income for the producer, they indirectly promote gains in subsequent crops. With the maintenance of soil cover, productivity gains and increased revenue are expected in production systems in the medium and long terms^21^. Cover crops, in general, control pests, diseases and weeds and improve soil conditions^36^ because they prevent soil compaction and improve soil water infiltration and retention, density, and hydraulic conductivity^45^. AS-III also had the lowest mean annual production cost; the cost with inputs was on average 35% lower than that observed in the other systems. The lower costs are because the cover crops were not harvested because their benefits are obtained from the biomass generated; thus, the cost is lower than that for systems for which the purpose is to sell grains. One of the great benefits of adopting this system is that the cultivation of cover crops in the winter can reduce the cost of the crop that follows because the amount of inputs involved in the production of the next crop can decrease, as can fuel expenses^46^. In addition, the lower demand for pesticides makes the system more economical and sustainable and less risky. The quantification and analysis of the items composing the costs of each system are extremely important for producers’ decision-making. However, this analysis requires extreme caution because higher production costs do not necessarily mean lower yields, and similarly, lower costs do not necessarily mean higher profits^20,21^.

AS-IV had the second lowest mean annual profit. This system included winter agroenergy crops. With the exception of canola, the other agroenergy crops grown in this production system showed low profitability. Despite having one of the lowest production costs, the low revenue obtained with agroenergy crops compromised the profitability of AS-IV. Even with the sale of crambe, safflower and canola, the revenues were not sufficient to cover the production costs. Although this system did not show one of the best results, studies with bioenergy crops are being conducted in various regions of the world, and these crops may become an option for southern Brazil, as in the case of Italy, where plants of the family Brassicaceae are being introduced in rotation with cereals as a source of income diversification^47^.

The lowest mean annual profit was observed for AS-I. The low profit is related to the high production costs. Despite having the second highest mean annual revenue, the high production cost compromised the profitability of the system. This result is associated with the lower grain productivity observed in this production system and the fact that it specialized in few crops and focused only on commodities, which are subject to changes in their sale price due to seasonality and market uncertainties, or with the increased susceptibility of this system to problems caused by climatic variations. The crops grown in this system are traded in the international market, and in this case, the producers are only “price takers,”, i.e., they are not able to influence the price of the products^48^. The prices of commodities may vary; thus, producers may obtain higher or lower revenue due to market fluctuations or volatility. In turn, market fluctuations or volatility are caused by, among other factors, production or external factors, such as exchange rate variations or increased food consumption^49,50^. AS-I had the highest mean annual pesticide costs, approximately 21% of the total cost (US$ 254). In addition to economic factors, the double-crop system has also generated problems such as the proliferation of pests, diseases and weeds because, in contrast to crop rotation, it does not interrupt the life cycles of pests and diseases^51^. To control the proliferation of pests, diseases and weeds, the increased use of inputs and an increase in the number of agricultural operations are required^52^, with a consequent increase in production costs^20^. This increase in production costs can be observed for winter corn crops, which were more expensive than summer soybean crops. In this system, the mean cost to produce soybean in the summer was US$ 567 per ha, and that to produce corn in the winter was US$ 648. Compared to the other systems studied, the average investment required for the winter growing season was US$ 448 and that for the growing season was US$ 640; that is, the winter crops required 30% less investment than the summer crops (Supplementary Table S3).

**Figure 3:**
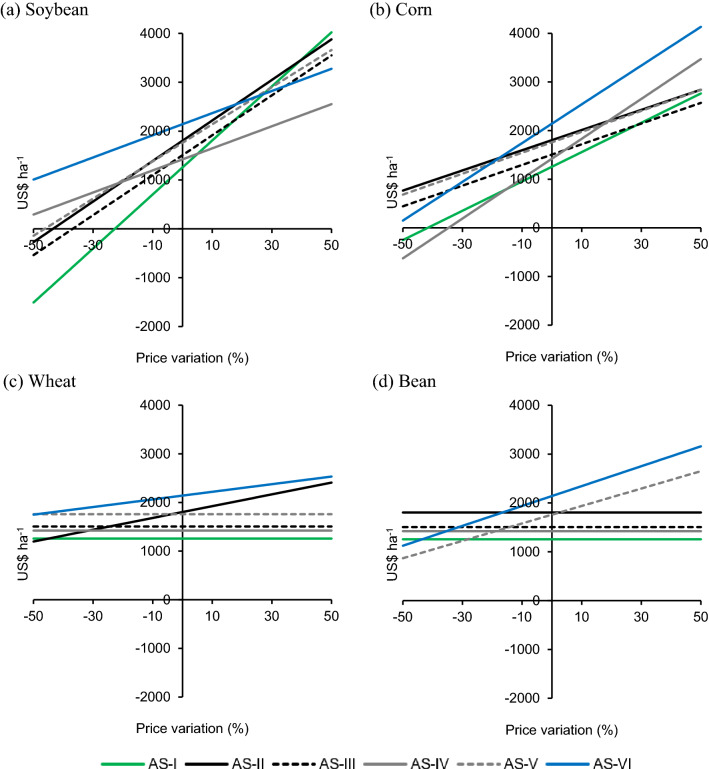
Price sensitivity analysis (accumulated profit of 6 crop years on the y-axis) of six agricultural systems in Londrina, state of Paraná, Brazil. (**a**) Soybean; (**b**) corn; (**c**) wheat; and (**d**) bean.

When considering the real selling price of grains, the highest accumulated profit was observed in AS-VI (Fig. 3); however, in a scenario in which the price of soybeans fluctuates (Fig. 3a) both upward and downward, sensitivity analysis revealed different behaviors. If there was a 44% increase in the selling price of soybeans, the ranking order of the systems would change, making AS-I more profitable. AS-I is the most sensitive to soybean price variations, since in this system, the crop is mainly responsible for generating income and is cultivated in all summers. Thus, the opposite results are also expected. A negative variation in the selling price of soybeans will make AS-I the system with the highest accumulated loss. Price changes can significantly increase or decrease the profitability of producers. Thus, the choice of crops and the number of times a crop appears in each agricultural system determines the profitability of the system as the sale price of the crops varies.

Corn showed some changes in the order of classification of the systems (Fig. 3b). If the corn sale prices were increased by up to 50%, AS-VI would continue to be the system with the highest accumulated profit. In this scenario, AS-I, composed solely of the corn crop in winter, would cease to be the system with the lowest accumulated profit, occupying the position of AS-III. Different from what happened with the soybean crop, the fluctuations in the corn sale price had less impact on AS-I in terms of accumulated profit. This was because the corn produced in this system accounted for a smaller share of profits and, in some cases, even resulted in losses.

Regarding the wheat crop (Fig. 3c), changes in the sale price led to little change in the accumulated profit. Wheat was grown only in AS-II and AS-VI, and in a scenario that considered only the variation in the price of this grain, if its selling price was reduced by up to 47%, AS-VI would continue to be the system with the highest accumulated profit. Changes in the selling price of the bean crop (Fig. 3d) had greater impacts. A 50% increase in the sale price of beans led to a 47% increase in profit in AS-VI.

**Figure 4:**
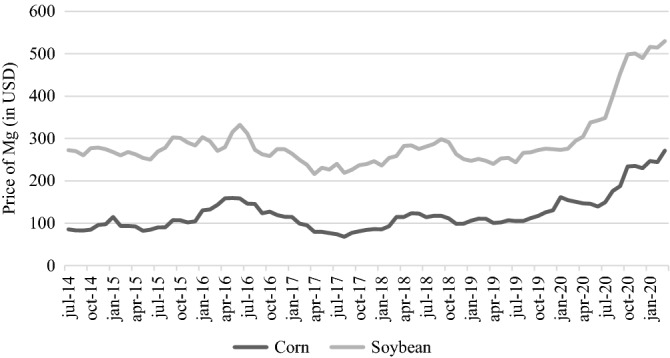
Evolution of corn and soybean prices from July 2014 to March 2020. Data were obtained from the Department of Rural Economy of the Paraná State Secretariat of Agriculture and Supply (DERAL-SEAB). The monetary values are corrected for inflation according to the Brazilian Extended National Consumer Price Index (IPCA) to December 2021.

In addition to variations in sale prices, another possible scenario is that crops are stored and sold at later dates. This is possible, as cooperatives are able to provide producers with storage and future sale of grains, extending the time for decision-making. Thus, producers can market products at an optimal time, e.g., when sale prices are better than those on the day of harvest. In this scenario, if corn and soybeans were stored and sold at peak prices recorded each quarter, over the 12 months following the harvest date, the evaluated agricultural systems would show even greater profits. Figure 4 shows the evolution of real prices in tons (USD) of corn and soybeans from July 2014 to March 2021.

If the sale of soybean and corn was carried out at times of price peaks, the accumulated profit of the systems would vary (Table 4). AS-I, composed exclusively of corn and soybean crops, would become the highest profit system (US$ 3,683). AS-VI, although no longer the highest profit system, would still be one of the systems with the best economic results (US$ 3479). In this scenario, AS-IV would occupy the last position, with the lowest accumulated profit (US$ 2732).

**Table 4:** Profit (USD ha^−1^) of the grain production systems for the 2014–2015 to 2019–2020 crop years, considering quarterly price peaks in Londrina, state of Paraná, Brazil. .

1st cycle						2nd cycle						Cumulative
2014–2015		2015–2016		2016–2017		2017–2018		2018–2019		2019–2020		
W	S	W	S	W	S	W	S	W	S	W	S	
**System I (AS-I)**												
C	S	C	S	C	S	C	S	C	S	C	S	
− 68	220	212	331	− 368	354	336	635	− 48	414	588	1077	3683
**System II (AS-II)**												
WO	S	RY	C	W	S	WO	S	TR	C	W	S	
− 133	393	− 377	600	− 95	456	− 28	675	− 122	410	113	1175	3068
**System III (AS-III)**												
BO + RY	S	BO + R	C	BR	S	BO + RY	S	BO + R	C	BR	S	
− 191	357	− 156	654	− 166	474	− 113	666	− 157	405	− 124	1167	2816
**System IV (AS-IV)**												
CL	C	CM	C	SF	S	CL	C	CM	C	CL	S	
125	452	− 278	599	− 174	421	− 244	436	− 216	390	− 21	1242	2732
**System V (AS-V)**												
BW/R	C	B	S	BW/WO	S	BW + R	C	B	S	BW/WO	S	
− 291	345	137	320	− 375	413	− 81	620	225	453	− 71	1087	2781
**System VI (AS-VI)**												
W	C + BR	CL	C	B	S	W	C	CL	C + BR	B	S	
− 199	360	− 145	635	56	474	− 36	402	− 60	271	463	1259	3479

*W* winter, *S* summer, *WO* white oat, *BO* black oat, *BR* brachiaria, *CL* canola, *CM* crambe, *RY* rye, *SF* safflower, *B* bean, *C* corn, *R* forage radish, *S* soybean, *W* wheat, *BW* buckwheat, *TR* triticale.

In this scenario, driven by the devaluation of the real against the dollar, the increase in domestic consumption and exports influenced the supply of grains in the market, and agricultural commodities such as soybeans and corn reached high sale values. Thus, it is evident that the market is able to condition the farmer's profitability, which can influence the results of the analysis, both positively and negatively, according to the daily variations in grain commercialization prices^53^.

From the results, it is evident that species diversification in crop rotation has enabled an increase in both grain productivity and economic gains. It is not enough to simply adopt no-till practices without species diversification in grain production systems^31,32^; it is necessary for the systems to be aligned with the no-tillage system and conservation agriculture principles. The main reasons for investing in crop diversification are as follows: production of roots and straw to cover the soil surface; improved soil structure and sustained soil biology; nutrient cycling; breaking the cycles of pests, diseases, and weeds; productivity gains; and increased profitability. Thus, the challenge lies in the diffusion of production systems aligned with the principles of the no-tillage system and conservation agriculture, that is, to diversify without failing to produce and obtain gains from grain production. Information on the benefits of grain production systems that employ crop rotation with species diversification, tested and with demonstrated economicity, such as those presented in this study, can therefore be decisive for producers’ decision-making and the adoption of practices aligned with sustainability in agriculture.

## Conclusion

Revenue and cost indicators cannot be analyzed in isolation. Profit is the main indicator to be considered for decision-making in agricultural production systems. This is because, although there is some relationship between productivity and profitability, the cropping systems with the best productivity and greater revenue were not necessarily the ones with the highest profits.

Compared to the double-cropped corn–soybean rotation, systems with species diversification showed better productivity and profitability. This was a result of the improved ecosystem services that conservation agriculture with more diverse crop rotations offered. The profit of the crop rotation systems with species diversification was on average 37% higher than that of the double-cropped corn‒soybean rotation, demonstrating the economic benefit of conservation agriculture.

In general, the production costs of winter crops were higher than those of summer crops, although the profitability and losses of the former were lower. The double-cropped corn–soybean system had higher costs in the winter than in the summer growing season, largely impacted by the costs of pesticides, while the opposite was observed for systems with diversified crop rotations.

Inputs represented the largest portion of the expenditures, corresponding to approximately 54% of the total cost, evidencing the importance of such a component for decision-making.

## Supplementary Information

Supplementary Information 1.

Supplementary Information 2.

Supplementary Information 3.

## Data Availability

All the data generated or analyzed during this study are included in this published article.
