# Supplementary material for: The diversification of species in crop rotation increases the profitability of grain production systems
Source: Sci Rep. 2022 Nov 18;12:19849. doi: 10.1038/s41598-022-23718-4 (PMC9674645; doi:10.1038/s41598-022-23718-4)
Supplement: Supplementary file 1 — Supplementary Information 1. [file 41598_2022_23718_MOESM1_ESM.pdf]

Crop	1st cycle						2nd cycle					
	**2014-2015**		**2015-2016**		**2016-2017**		**2017-2018**		**2018-2019**		**2019-2020**	
	**W**	**S**	**W**	**S**	**W**	**S**	**W**	**S**	**W**	**S**	**W**	**S**
Maize	83.12	93.40	85.14	130.56	135.67	-	66.12	100.70	90.30	89.31	102.07	-
Soybean	-	255.73	-	277.20	-	262,92	-	249.65	-	232.21	-	281.07
Wheat	141.07	-	-	-	154.37	-	129.43	-	-	-	164.25	-
Beans	-	-	447.38	-	1,383.19	-	483.85	-	411.72	-	-	-
Buckwheat	147.33	-	-	-	155.85	-	147.65	-	-	-	-	-
Canola	266.10	-	261.79	-	-	-	226.30	-	-	-	-	-
Crambe	-	-	139.09	-	-	-	-	-	-	-	-	-
Safflower	-	-	-	235.56	-	-	-	-	-	-	-	-
Triticale	-	-	-	-	-	-	-	-	118.73	-	-	-

Supplementary Table S1. Price (USD Mg^{-1}) of the harvested and marketed crops for the 2014-2015 to 2019-2020 crop years in Londrina, state of Paraná, Brazil. "-": not grown in that crop year. W: winter; S: summer. Data from the Department of Rural Economy of the Paraná State Secretariat of Agriculture and Supply (DERAL-SEAB). Monetary values corrected for inflation according to the Brazilian Extended National Consumer Price Index (IPCA), to December 2021.
