# Supplementary material for: The diversification of species in crop rotation increases the profitability of grain production systems
Source: Sci Rep. 2022 Nov 18;12:19849. doi: 10.1038/s41598-022-23718-4 (PMC9674645; doi:10.1038/s41598-022-23718-4)
Supplement: Supplementary file 2 — Supplementary Information 2. [file 41598_2022_23718_MOESM2_ESM.pdf]

Indicator	1st cycle						2nd cycle					
	2014-2015		2015-2016		2016-2017		2017-2018		2018-2019		2019-2020	
	Winter	Summer	Winter	Summer	Winter	Summer	Winter	Summer	Winter	Summer	Winter	Summer
**System I (AS-I)**												
	C	S	C	S	C	S	C	S	C	S	C	S
Revenue	490	758	556	898	371	1,060	532	1,033	521	763	545	1,019
Cost	692	634	704	639	607	625	618	525	776	465	491	513
Profit	-202	124	-147	259	-236	434	-87	508	-255	298	54	506
**System II (AS-II)**												
	WO	S	RY	C	W	S	WO	S	TR	C	W	S
Revenue	322	900	- -	1,288	581	1,097	336	1,066	404	786	631	1,086
Cost	455	620	377	782	676	634	364	521	526	700	518	520
Profit	-133	279	-377	506	-96	464	-27	544	-123	86	113	566
**System III (AS-III)**												
	BO+RY	S	BO+R	C	BR	S	BO+RY	S	BO+R	C	BR	S
Revenue	- -	877	- -	1,345	- -	1,075	- -	1,056	- -	782	- -	1,081
Cost	191	631	156	789	166	641	113	520	157	700	124	521
Profit	-191	247	-156	556	-166	434	-113	536	-157	83	-124	561
**System IV (AS-IV)**												
	CL	C	CM	C	SF	S	CL	C	CM	C	CL	S
Revenue	467	1,046	205	1,208	237	1,128	83	1,073	- -	771	253	1,132
Cost	342	734	483	697	411	647	327	827	216	698	274	525
Profit	125	312	-278	511	-174	481	-244	245	-216	73	-21	607
**System V (AS-V)**												
	BW/R	C	B	S	BW/WO	S	BW+R	C	B	S	BW/WO	S
Revenue	171	993	888	833	122	1,143	244	1,158	896	801	245	1,026
Cost	462	781	751	580	497	648	325	745	671	470	316	514
Profit	-291	211	136	254	-375	494	-81	414	224	331	-71	512
**System VI (AS-VI)**												
	W	C+BR	CL	C	B	S	W	C	CL	C+BR	B	S
Revenue	259	984	299	1,252	1,073	1,125	524	1,041	303	707	962	1,143
Cost	458	755	444	709	1,017	647	560	825	363	727	499	526
Profit	-199	229	-146	543	56	479	-36	217	-60	-20	463	618

Supplementary Table S2. Revenue, cost and profit (US$ ha^{-1}) of the grain production systems for the 2014-2015 to 2019-2020 crop years. WO: white oat, BO: black oat, BR: brachiaria, CL: canola, CM: crambe, RY: rye, SF: safflower, B: bean, C: corn, R: forage radish, S: soybean, W: wheat, BW: buckwheat, TR: triticale.
