# Supplementary material for: The diversification of species in crop rotation increases the profitability of grain production systems
Source: Sci Rep. 2022 Nov 18;12:19849. doi: 10.1038/s41598-022-23718-4 (PMC9674645; doi:10.1038/s41598-022-23718-4)
Supplement: Supplementary file 3 — Supplementary Information 3. [file 41598_2022_23718_MOESM3_ESM.pdf]

Indicator	1st cycle						2nd cycle					
	2014-2015		2015-2016		2016-2017		2017-2018		2018-2019		2019-2020	
	Winter	Summer	Winter	Summer	Winter	Summer	Winter	Summer	Winter	Summer	Winter	Summer
**System I (AS-I)**												
	C	S	C	S	C	S	C	S	C	S	C	S
Total cost	692	634	704	639	607	625	618	525	776	465	491	513
Inputs	427	292	413	282	395	326	388	258	533	241	293	261
Seeds	125	76	130	51	104	109	163	75	145	66	106	95
Fertilizers	169	79	170	84	158	75	124	67	152	56	121	88
Pesticides	133	137	112	147	133	142	102	116	236	118	66	78
Agric. operations	179	249	201	257	139	193	148	171	148	145	127	157
Other costs	86	93	90	100	73	106	81	96	95	78	70	94
**System II (AS-II)**												
	WO	S	RY	C	W	S	WO	S	TR	C	W	S
Total cost	455	620	377	782	676	634	364	521	526	700	518	520
Inputs	262	292	252	381	371	326	170	258	340	409	301	261
Seeds	78	76	77	103	97	109	43	75	38	103	43	95
Fertilizers	131	79	152	167	135	75	53	67	164	194	141	88
Pesticides	54	137	24	111	140	142	73	116	137	112	116	78
Agric. operations	136	230	90	271	217	199	145	166	119	191	140	160
Other costs	57	98	35	131	89	109	49	97	67	100	77	98
**System III (AS-III)**												
	BO+RY	S	BO+R	C	BR	S	BO+RY	S	BO+R	C	BR	S
Total cost	191	631	156	789	166	641	113	520	157	700	124	521
Inputs	82	274	63	381	62	326	37	258	85	409	29	263
Seeds	38	58	39	103	30	109	25	75	30	103	13	95
Fertilizers	0	79	0	167	0	75	0	67	0	194	0	88
Pesticides	44	137	24	111	32	142	12	116	55	112	16	80
Agric. operations	90	258	78	274	88	207	64	166	57	191	83	160
Other costs	18	98	15	134	16	108	11	97	15	100	12	98
**System IV (AS-VI)**												
	CL	C	CM	C	SF	S	CL	C	CM	C	CL	S
Total cost	342	734	483	697	411	647	327	827	216	698	274	525
Inputs	170	404	265	360	241	326	208	484	138	409	155	261
Seeds	4	147	56	97	31	109	77	111	18	103	6	95
Fertilizers	131	167	172	167	142	75	104	159	97	194	133	88
Pesticides	35	90	37	96	68	142	27	214	23	112	16	78
Agric. operations	119	215	164	217	121	210	84	218	57	189	81	163
Other costs	53	115	54	119	49	111	34	125	21	100	37	101
**System V (AS-V)**												
	BW/R	C	B	S	BW/WO	S	BW/R	C	B	S	BW/WO	S
Total cost	462	781	751	580	497	648	325	745	671	470	316	514
Inputs	261	424	410	268	293	326	129	399	444	241	131	261
Seeds	63	166	137	51	99	109	101	107	139	66	102	95
Fertilizers	112	167	152	84	132	75	0	159	164	56	0	88
Pesticides	87	90	121	133	61	142	28	133	141	118	29	78
Agric.operations	150	240	233	220	153	211	154	224	124	148	144	157
Other costs	50	117	109	92	51	112	42	122	103	80	41	95
**System VI (AS-VI)**												
	W	C+BR	CL	C	B	S	W	C	CL	C+BR	B	S
Total cost	458	755	444	709	1.017	647	560	825	363	727	499	526
Inputs	277	431	233	366	705	326	385	399	199	397	262	261
Seeds	93	176	5	103	289	109	113	107	4	116	20	95
Fertilizers	131	167	172	167	158	75	111	159	141	194	121	88
Pesticides	54	87	56	96	259	142	162	133	54	87	121	78
Agric.operations	127	210	157	220	170	210	99	302	116	231	147	163
Other costs	54	114	55	123	142	111	76	123	48	99	91	101

Supplementary Table S3. Production cost of the grain production systems for the 2014-2015 to 2019-2020 crop years (in US$ ha^{-1}). WO: white oat, BO: black oat, BR: brachiaria, CL: canola, CM: crambe, RY: rye, SF: safflower, B: bean, C: corn, R: forage radish, S: soybean, W: wheat, BW: buckwheat, TR: triticale.
